# Changes in immune cell signatures during early infection reflect decoupling of capillary perfusion and glycocalyx dimensions

**DOI:** 10.3389/fimmu.2025.1589959

**Published:** 2025-05-23

**Authors:** Anna Hunkemöller, Timo Wirth, Alexandros Rovas, Hermann Pavenstädt, Luisa Klotz, Philipp Kümpers

**Affiliations:** ^1^ Department of Medicine D, Division of General Internal and Emergency Medicine, Nephrology, and Rheumatology, University Hospital Münster, Münster, Germany; ^2^ Department of Neurology with Institute of Translational Neurology, University Hospital Münster, Münster, Germany

**Keywords:** sepsis, microcirculation, sublingual microscopy, endothelial glycocalyx, immune phenotypes, flow cytometry

## Abstract

**Introduction:**

Microvascular injury is central to the pathophysiology of sepsis, but its interaction with the immune system in early infection is unclear. This study aimed to phenotype peripheral blood mononuclear cells (PBMC) from emergency department (ED) patients with suspected bacterial infection and correlate the results with microvascular changes.

**Methods:**

This prospective observational study included 49 adult ED patients with suspected infection and 17 healthy controls. Capillary density and glycocalyx dimensions were measured by sublingual microscopy, while peripheral blood immune cell subsets were analyzed by deep flow cytometry.

**Results:**

Network visualization of 72 diIerentially regulated parameters revealed specific changes in diIerent immune cell subsets. Innate immune changes included a functional diversion of monocytes towards pathogen defense and tissue repair, whereas adaptive immune changes included the development of CD4+ T cells with Th2-profile and cytotoxic CD8+ T cells. Unsupervised clustering revealed two distinct immune endotypes: E1 with a suppressed immune response and higher disease severity, and E2 with an enhanced immune response and lower disease severity. Patients showed significant reductions in capillary density and glycocalyx dimensions, which were neither correlated in magnitude nor associated with endotypes. There was a strong association between damaged glycocalyx and several monocyte and T-cell subsets. This association was not observed for capillary density.

**Discussion:**

We demonstrate that glycocalyx damage is associated with a unique immunological signature, distinct from functional capillary density. These findings provide a strong basis for future studies of immune dysregulation and microvascular dysfunction in infection.

## 1 Introduction

Sepsis is a complex and life-threatening condition characterized by organ dysfunction due to a dysregulated host response to systemic infection. It is associated with an in-hospital mortality rate of over 10% and remains a leading cause of death in intensive care units worldwide (1, 2).

The pathophysiology of sepsis involves an imbalance between pro- and anti-inflammatory processes, impaired endothelial function and increased blood coagulation, which together impair microcirculation and lead to capillary leakage and ultimately multi-organ failure (3, 4).

Although the host response to infection in severe sepsis and septic shock has been extensively studied, few investigations have focused on the early phase of infection.

Using quantitative analysis of sublingual non-invasive sidestream darkfield (SDF) imaging, our previous translational research has shown that microcirculatory disturbances are among the earliest events in the host response, alongside cytokine release (5, 6).

Specifically, systemic inflammation has been demonstrated to disrupt the endothelial glycocalyx (eGC), impair capillary perfusion and reduce functional microvascular density (6–8). The eGC is an up to 3 µm thick delicate gel-like layer of highly sulfated glycosaminoglycans and proteoglycans, coating the luminal surface of the entire vascular endothelium (9, 10). It protects the endothelium from leukocyte adhesion and hyperpermeability, is critical in mediating shear-induced nitric oxide release and contributes to physiologic anticoagulation (11, 12). We have shown that specific cytokine signatures reflect eGC damage (13) and that interleukin 6 (IL-6) plays a causal role in glycocalyx damage in both patients with coronavirus disease 2019 (COVID-19) and those with bacterial sepsis (14).

These findings prompted us to investigate the cellular and immunological processes that precede cytokine release, thereby enhancing our comprehension of the host response to infectious stimuli. The aim of this study was to perform a comprehensive phenotyping of peripheral blood mononuclear cells (PBMC) from patients presenting to the Emergency Department (ED) with an infection and to correlate the observed changes with cytokine and microcirculatory alterations.

## 2 Materials and methods

### 2.1 Study design and study population

This prospective, observational study was conducted between December 2021 and June 2022 in the interdisciplinary ED of the University Hospital of Münster. The study adhered to the Declaration of Helsinki and was approved by the Ethics Committee (2016–073-f-S and amendments). A total of 49 adult patients with clinically suspected bacterial infection requiring intravenous antibiotic treatment and anticipated hospitalization were prospectively enrolled nonconsecutively after initial triage. Written informed consent was obtained from either the patients or their legal representatives. Sepsis was defined according to Sepsis-3 criteria (1). Exclusion criteria were age < 18 years, pregnancy, current or recent malignancy or chemotherapy, current or recent use of immunosuppressive drugs (e.g. prednisone, methotrexate, mycophenolate, immune checkpoint inhibitors), and oral mucosal injury or inflammation that could affect the sublingual microvasculature. A total of 17 healthy volunteers (age ≥ 18 years) were recruited to serve as controls.

For each subject, demographic, laboratory, and physiological variables and scores, including the Sequential Organ Failure Assessment (SOFA) score (1), the Modified Early Warning Score (MEWS) (15), the Systemic Inflammatory Response Syndrome (SIRS) criteria (16), and a contemporary version of the Charlson Comorbidity Index (CCI) (17), were collected and computed at the time of sublingual videomicroscopy (**Table 1**). Blood samples were pseudonymized prior to storage and subsequent analysis, with a portion cryopreserved for flow cytometry measurements (18).

**Table 1:** Baseline characteristics.

Variable	Healthy volunteers	Patients with suspected infection			
Demographic variables		Total	E1	E2	P_E1 vs. E2_
Number of participants (n (%))	17 (25.8)	49 (74.2)	22 (44.9)	27 (55.1)	–
Female sex (n (%))	12 (70.6)	15 (30.6)	5 (22.7)	10 (37.0)	0.358
Age (years, median (IQR))	57 (39 – 60)	72 (58 – 82)	76 (68 – 84)	64 (48 – 79)	0.040
BMI (kg/m^2^, median (IQR))	25.2 (22.6 – 27.1)	28.4 (23.7 – 31.2)	27.7 (23.6 – 30.2)	30.1 (24.4 – 32.9)	0.172
CCI score updated (median (IQR))	–	2 (0 – 4)	3 (1 – 4)	1 (0 – 3)	0.006
Clinical variables					
Sepsis (according to S-3 definition) (n (%))	–	25 (51.0)	13 (59.1)	11 (40.7)	0.256
SOFA score (median (IQR))	–	2 (1 – 4)	3 (2 – 5)	1 (0 – 3)	0.005
SIRS	–	1 (1 – 2)	2 (1 – 3)	1 (0 – 2)	0.057
MEWS	–	3 (2 – 6)	5 (3 – 8)	3 (0 – 6)	0.033
Focus of infection (n (%))					0.298
Gastrointestinal	–	13 (26.5)	6 (27.3)	7 (25.9)	
Respiratory tract	–	11 (22.4)	7 (31.8)	4 (14.8)	
Urinary tract	–	10 (20.4)	4 (18.2)	6 (22.2)	
Other	–	9 (18.3)	8 (36.3)	5 (18.5)	
Unknown	–	6 (12.2)	1 (4.5)	5 (18.5)	
Sublingual microscopy (median (IQR))					
Capillary density 4-7 µm (10^–2^ mm/mm^2^)	96.9 (70.0 – 109.6)	71.2 (52.5 – 86.8)	72.8 (53.4 – 83.3)	69.0 (51.7 – 90.7)	0.968
PBR 4-25 μm (μm)	1.94 (1.81 – 2.12)	2.28 (2.13 – 2.44)	2.32 (2.16 – 2.44)	2.24 (2.09 – 2.43)	0.445
MVHS (pts)	3.9 (2.9 – 4.6)	2.2 (1.6 – 2.8)	2.1 (1.5 – 2.6)	2.3 (1.6 – 2.9)	0.198
Laboratory data (median (IQR))					
WBC (10^3^ cells/μl)	5.960 (4.280 – 6.750)	12.910 (10.150 – 17.255)	15.040 (11.198 – 17.975)	11.660 (9.350 – 15.020)	0.044
Thrombocytes (10^3^/μl)	241 (213 – 304)	206 (143 – 261)	214 (156 – 281)	201 (134 – 240)	0.560
Creatinine (mg/dl)	0.8 (0.7 – 0.9)	1.4 (1.0 – 2.3)	1.8 (1.0 – 3.0)	1.2 (0.8 – 1.9)	0.068
Bilirubin (mg/dl)	0.4 (0.2 – 0.8)	0.9 (0.5 – 1.2)	1.0 (0.5 – 1.3)	0.8 (0.5 – 1.1)	0.330
CRP (mg/dl)	0.5	10.2 (7.1 – 22.0)	20.3 (8.1 – 31.2)	7.6 (4.2 – 14.4)	0.004
IL-6 (pg/ml)	2.0	80 (36 – 226)	119 (38 – 709)	56 (26 – 138)	0.083
PCT (ng/ml)	0.02 (0.02 – 0.03)	0.74 (0.16 – 4.08)	2.1 (0.6 – 14.2)	0.4 (0.1 – 1.1)	< 0.001
Lactate (mmol/l)	1.1 (0.8 – 1.6)	1.2 (0.9 – 1.5)	1.4 (0.95 – 1.7)	1.0 (0.8 – 1.3)	0.032
Outcome parameters					
Clinical progression (n (%))	–	13 (26.5)	8 (36.4)	4 (18.2)	0.104
30-day mortality (n (%))	–	4 (8.2)	2 (9.1)	2 (7.4)	1.000

E, Endotype; BMI, body mass index; CCI score, Charlson Comorbidity Index score; CRP, C-reactive protein; IL-6, interleukin-6; IQR, interquartile range; MAP, mean arterial pressure; PBR, perfused boundary region; PCT, procalcitonin; SOFA score, Sequential Organ Failure Assessment score; WBC, white blood cells.

### 2.2 Analysis of sublingual microvasculature

A sidestream dark field camera (CapiScope HVCS, KK Technology, Honiton, UK) coupled with GlycoCheck™ software (Microvascular Health Solutions Inc., Salt Lake City, Alpine, UT, USA) was used to visualize red blood cells (RBC) flow in the sublingual microvasculature (microvessel diameter 4–25 µm) at the bedside as previously described in detail (6, 8). Based on the RBC dynamics in the valid vascular segments, the software calculates the following variables, which were successfully validated in the past (7, 8, 19):

*Perfused boundary region* (PBR, in µm) estimates the dynamic lateral movement of the RBCs into the permeable part of the endothelial glycocalyx layer. Impaired eGC allows RBCs to penetrate deeper into the endothelium, resulting in higher PBR values.

*Capillary density* (10⁻² mm/mm²) was calculated by multiplying the number of RBC-containing vessel segments by the segment length (10 µm each) normalized to the tissue surface area. Capillary density hereafter refers to the combined density of capillaries with diameters between 4 and 7 µm (D4−7 µm).

*The Microvascular Health Score* (MVHS; in points) is a composite metric, which has been previously presented in detail (7). It integrates glycocalyx dimensions (PBR) and capillary density into a single parameter, which serves as a combined surrogate marker for microvascular integrity.

**Figure 1:**
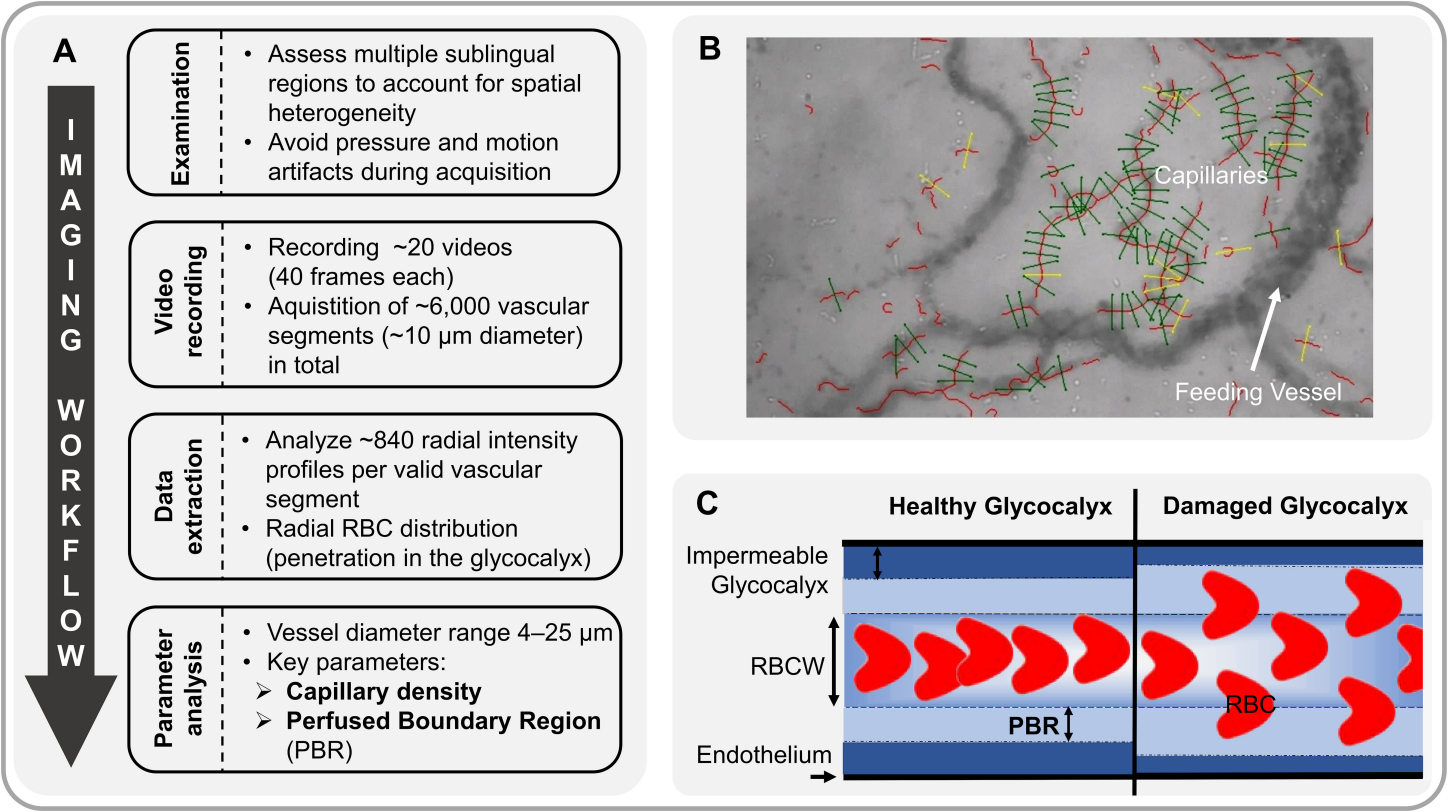
Analysis of sublingual microvasculature. **(A)** Workflow of sublingual video acquisition and analysis of microvascular parameters. **(B)** Sidestream Dark Field (SDF) image showing software-identified valid (green) and invalid (yellow) capillaries (vascular segments). **(C)** Schematic of red blood cell (RBC) flow through a capillary under healthy conditions (left). The perfused boundary region (PBR, in µm) estimates this dynamic lateral movement of RBCs into the permeable part of the endothelial glycocalyx layer. Impaired glycocalyx allows RBCs to penetrate deeper into the endothelium, resulting in higher PBR values (right). RBCW, Red Blood Cell Column Width.

**Figure 1** provides an overview of the process of video acquisition, data analysis and post-processing.

### 2.3 Processing of PBMC

Cryopreserved PBMC were thawed in a 37°C water bath for 8 min, resuspended in 9 ml pre-warmed RPMI medium (RPMI (Sigma Aldrich, St. Louis, USA), 10% FCS Gold Plus (BioSell, Feucht, Germany), 1% Glutamax (Gibco, Paisley, Great Britain), 1% Na-pyruvate (Invitrogen, Carlsbad, USA)) and centrifuged at 300 g for 10 min at room temperature. The supernatant was discarded, and the cell pellet was resuspended in RPMI medium. PBMCs were counted and viability assessed using a Countess II automated cell counter (Invitrogen, Carlsbad, USA). Intracellular staining, surface staining and functional assays were performed, followed by flow cytometric analysis.

### 2.4 Flow cytometry measurements

PBMC were analyzed by flow cytometry as previously described (18). **Supplementary Tables 1**-**3** detail the gating strategies, antibodies and fluorochromes used. Briefly, for surface molecule staining, cells were washed with flow cytometry buffer (a buffer supplemented with 2% heat-inactivated fetal bovine serum (FBS) and 2 mM ethylenediaminetetraacetic acid (EDTA, Sigma-Aldrich)) and incubated with the appropriate antibodies for 30 minutes at either 4°C or 37°C. True Stain Monocyte Blocker™ (BioLegend, San Diego, USA) was added for monocyte staining according to the manufacturer’s instructions. When indicated, PBMC were additionally stained with Zombie NIR™ to eliminate dead cells or streptavidin (both BioLegend).

For intracellular staining, cells were additionally fixed with Perm/Fix buffer (BD Biosciences, San Jose, USA) for 20 min at room temperature, followed by staining in Perm buffer (BD Biosciences) for 30 min at 4°C. Finally, the functional capacity of immune cell subsets was assessed by both non-specific stimulation with PMA/ionomycin/brefeldin A (Leukocyte Activation Cocktail, LAC, BD Biosciences) and specific stimulation by redirected cross-linking of CD3 or DNAM1 and 2B4, followed by staining for lineage markers and cytokines or CD107a as a marker for directed degranulation of cytolytic vesicles. Monocyte cytokine production was induced by stimulation with 100 ng/ml LPS (E. coli O26:B6, Sigma-Aldrich) at 37°C with 5% CO2 for 2 h. Samples were collected on a Cytoflex S flow cytometer (Beckman Coulter, Brea, USA) with daily quality control using CytoFlex Daily QC Fluorospheres (Beckman Coulter). Flow cytometric data were analyzed using Kaluza 2.1 software (Beckman Coulter) by manual gating on established PBMC subsets and on markers of degranulation of cytolytic vesicles or cytokines.

### 2.5 Statistical analysis

Data are presented as absolute numbers, percentages, and medians with corresponding 25th and 75th percentiles (interquartile ranges; IQRs) where appropriate. Nonparametric Mann-Whitney U and Fisher exact tests were used to compare groups, as appropriate. Spearman’s correlation coefficient was used to assess correlations between variables. Regulated markers associated with infection were visualized using the volcano plot, which visualizes the p values derived from the testing of a respective parameter and the corresponding log_2_ fold change (FC) of the medians. All tests used were two-tailed and statistical significance was set at p < 0.05. Statistical analyses of clinical variables were performed using SPSS version 29 (IBM Corporation, Armonk, USA) and GraphPad Prism version 10.1.1 (Graph Pad Prizm Inc., San Diego, USA).

### 2.6 Network visualization

Differences between groups or correlation coefficients across cellular networks were plotted as networks using Cytoscape version 3.10.2 (ISB, Seattle, USA). For this purpose, tables with nodes, edges, significance labels, and log_2_ FC values were imported, and information was illustrated by distinct border line types and fill colors.

### 2.7 Uniform Manifold Approximation and Projection and PhenoGraph

R environment for statistical computing (Version 4.1.2, R Foundation for Statistical Computing, Vienna, Austria) were used for data analysis and graph generation. Dimensionality reduction technique Uniform Manifold Approximation and Projection (UMAP) implemented in the R package “uwot” (0.1.14) was used to visually summarize the multidimensional structure of the data characterizing each individual participant as described previously (18). The PhenoGraph algorithm implemented in the R package “Rphenograph” (0.99.1) was applied for automated grouping (clustering) based on high-dimensional characteristics of the respective data (18). Resulting p values were corrected for multiple testing by the Benjamini-Hochberg false discovery rate (FDR) method, resulting in the adjusted q values.

### 2.8 Least Absolute Shrinkage and Selection Operator

**Figure 2:**
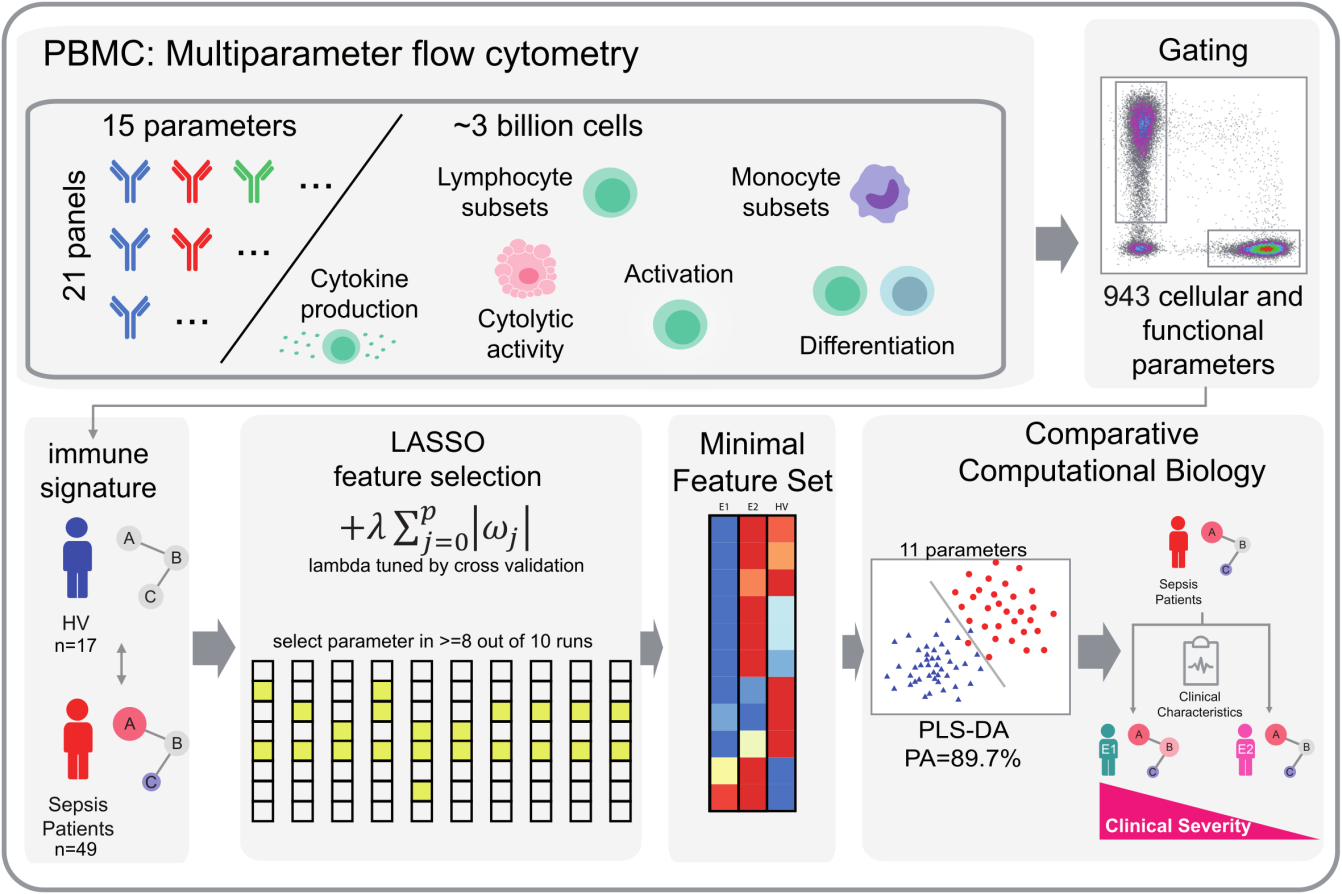
Workflow of LASSO-based feature selection and PLS-DA classification analysis. Our analytical pipeline involved a two-step approach, combining LASSO-based feature selection with PLS-DA classification analysis. We began by generating comprehensive flow cytometry datasets, comprising 943 cellular and functional parameters that served as individual peripheral immune signatures. To identify robust parameters that distinguish immunological endophenotypes, we applied a LASSO-based feature selection procedure. Specifically, we split our dataset into 10 subsets and performed LASSO regularization with multinomial logistic regression on 9 subsets per iteration, using 10-fold random subsampling to evaluate model performance. We tuned the regularization parameter λ using inner cross-validation. By repeating this process 10 times, we obtained 10 sets of selected parameters, from which we retained only those parameters that were consistently selected in at least 8 out of 10 iterations. This approach allowed us to trade off some prediction accuracy for increased robustness and reduced the dimensionality of our dataset. The resulting feature set was then subjected to PLS-DA. We visualized the first two dimensions of the PLS-DA results to illustrate group separation based on the identified feature sets. HV, Healthy Volunteers.

To identify parameters that robustly contribute to the differentiation of infection endophenotypes, we used a two-step Least Absolute Shrinkage and Selection Operator (LASSO) focused analysis as described previously (18). To this end, each dataset was randomly divided into 10 subsets. We then performed LASSO regularization, evaluated by multinomial logistic regression using the “glmnet” package (4.1-3) in R (20), on 8 out of 10 in each run, with lambda tuned by internal cross-validation. We selected only those parameters that were selected in at least 8 out of 10 parameter sets to increase robustness of parameter selection (**Figure 2**).

### 2.9 Partial least-squares discriminant analysis

Partial least-squares discriminant analysis (PLS-DA) was used to describe the separation of groups identified by LASSO. The plsda function from the “mixOmics” package (6.24.0) was used in RStudio (1.1.442) running R4.0.2. The first two dimensions were plotted.

## 3 Results

### 3.1 Clinical characteristics

Our data set consisted of 49 adult patients with a median age [IQR] of 72 [58 - 82] years. The majority of patients were male (n = 34; 69%), overweight (median BMI: 28.4 [23.7 – 31.2] kg/m^2^), and had a median SOFA score of 2 [1 – 4] points. 24.5% experienced clinical deterioration within 7 days and 30-day mortality was 8.2%. The control group consisted of 17 healthy volunteers (age [IQR]: 57 [39 – 60], 71% female). Detailed demographic and clinical characteristics are shown in **Table 1**.

### 3.2 Immunological signature changes in ED patients with infection

**Figure 3:**
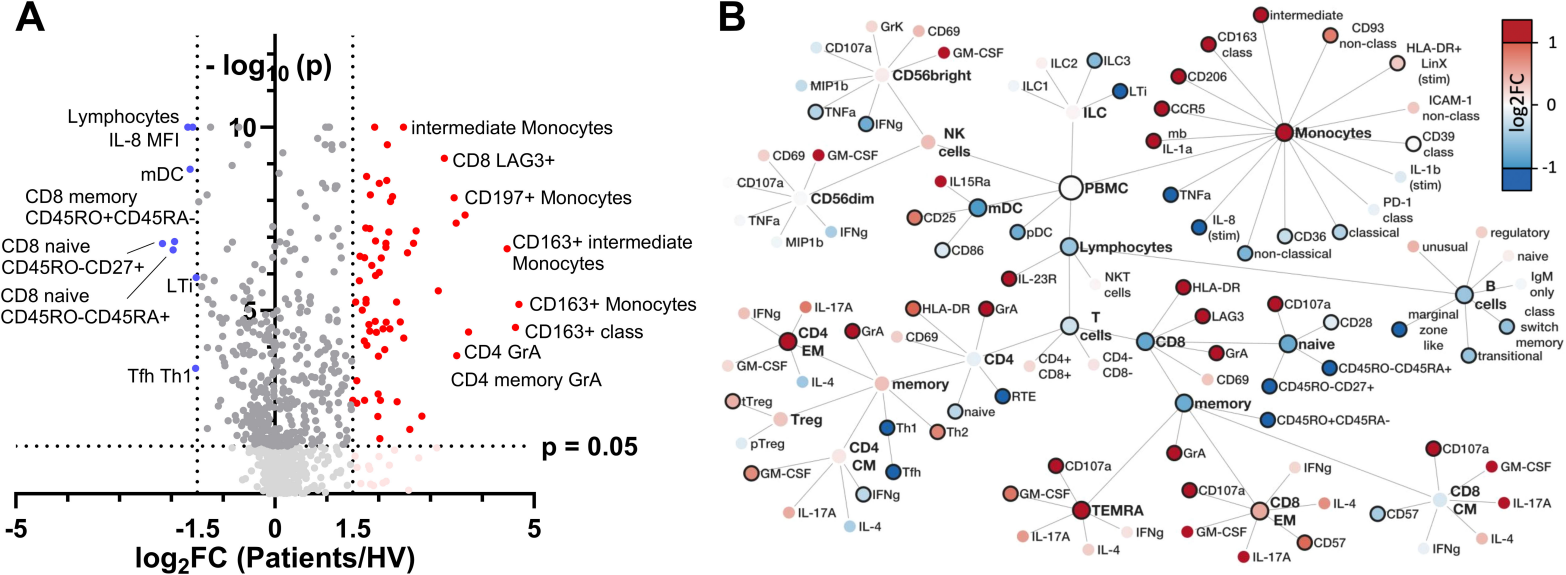
Identification and hierarchical analysis of immune markers. **(A)** Volcano plot displaying the log_2_ FC of immune markers between infected and healthy individuals, highlighting significantly upregulated (red) and downregulated (blue) markers based on p value thresholds. **(B)** Hierarchical network representation of selected markers, organized by their involvement in innate and adaptive immunity, with color intensity reflecting the magnitude of log_2_ FC and solid node borders indicating statistical significance with p < 0.05. FC, fold change; mb, membranous; stim, stimulated with LPS; LTi, lymphoid tissue inducer.

A total of 72 differentially regulated parameters were identified in the volcano plot analysis of 943 discrete immune cell subsets, cell surface markers, cytokines, chemokines, cytolytic granule content and cytolytic activity (hereafter cellular signatures) measured by flow cytometry. Of these, 64 were significantly up-regulated and 8 were significantly down-regulated at a log_2_ FC threshold of +/- 1.5 in the patients with infection (**Figure 3A**).

Network visualization of differentially regulated parameters highlighted complex and potentially integrated alterations in both innate and adaptive immune cell signatures during early infection (**Figure 3B**).

Considering the innate immune compartment (**Figure 3B** upper part), a pronounced shift in monocyte subsets was observed, with specific markers indicating an upregulation of both markers of pathogen defense and tissue repair. CCR5 upregulation on monocytes suggests enhanced chemotaxis, possibly facilitating monocyte recruitment to infection sites. Concurrently, CD206 expression aligns with an M2-like phenotype, favoring phagocytosis and tissue repair activities, alongside anti-inflammatory regulation by CD163 expression on classical (CD14+CD16-) monocytes, potentially modulated by CD121b expression.

Myeloid dendritic cells (mDCs) were less abundant in sepsis patients, while upregulation of CD25 and CD86 on their surface suggest an immunoregulatory capacity. Natural killer (NK) cells exhibited signs of exhaustion evident through diminished TNF-α and IFN-γ production. Furthermore, a notable reduction in distinct innate lymphoid cell (ILC) populations, particularly lymphoid tissue inducer (LTi) cells and ILC3 subsets, suggests a shift in innate cell-mediated responses. This profile highlights a finely tuned innate immune response, balancing antimicrobial defense, tissue repair, and immunoregulatory functions.

In adaptive immune compartments (**Figure 3B** bottom part), CD4 T cells in sepsis patients showed an increase of highly activated, cytotoxic CD4 T cells (HLA-DR, GrA) and significant increase in abundance of CD4 effector memory cells in conjunction with a shift towards a Th2-directed adaptive immune profile. Albeit CD8 T cell abundances in total were decreased in sepsis patients, CD8 memory subset composition was shifted from central memory (CM) towards an increase in CD8 effector memory (EM) and CD8 TEMRA cells. Furthermore, CD8 EM cells showed increased expression of molecules consistent with a cytotoxic phenotype (CD107a, HLA-DR) and incipient exhaustion (LAG3). B cells decreased globally, in conjunction with a decline in transitional and memory subsets.

Overall, ED patients presenting with infection show a complex cellular signature shift characterized by both activation and suppression within different immune cell subsets.

### 3.3 Identification of discrete immunological endophenotypes

**Figure 4:**
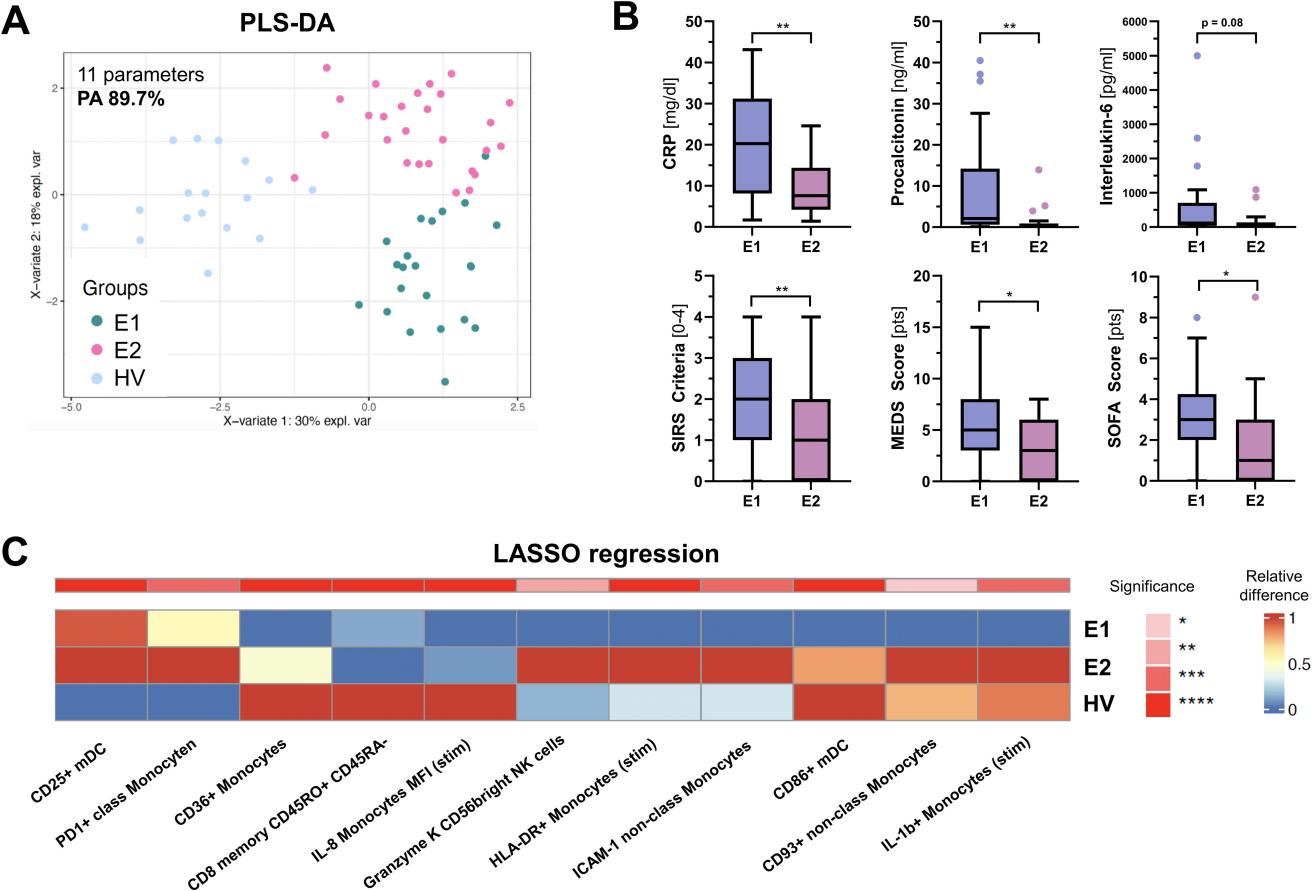
PLSDA analysis of PBMC parameters identified by LASSO. **(A)** shows a PLS-DA (Partial Least Squares Discriminant Analysis) score plot based on 11 PBMC parameters identified by LASSO (Least Absolute Shrinkage and Selection Operator) in at least 8 out of 10 runs. These parameters effectively distinguish between the three groups: Endophenotype 1 (E1), Endophenotype 2 (E2), and Healthy volunteers (HV), with a prediction accuracy (PA) of 89.7%. **(B)** Box and whisker plots showing differences in clinical parameters and severity scores between E1 and E2. **(C)** displays heatmaps of the relative median frequency for each parameter, where color intensity represents the expression level and significance across the groups. Red signifies upregulation, blue denotes downregulation, and asterisks indicate statistical significance based on the Kruskal-Wallis test, corrected for multiple comparisons. “stim” = stimulated with Lipopolysaccharides (LPS). *p < 0.05, ** p < 0.001.

To further elucidate the immunological profile in ED patients and its potential relevance for the clinical outcome, we sought to identify potential heterogeneities in immune response profiles and explore whether such variations could be indicative of discrete disease subtypes. Unsupervised cluster analysis of all immunological parameters measured by flow cytometry not only separated ED patients from HV, but identified two discrete immunological ED endotypes, each with distinct patterns of cellular immune signatures (**Supplementary Figure 1**). By using LASSO regression as a feature selection method, we isolated a small group of 11 subgroup-specific parameters capable of achieving a high prediction performance, with an overall predictive accuracy (PA) of 89.7% for endophenotype (E) differentiation (**Figures 4A, B**). Compared to healthy volunteers, E1 (n = 22) showed a dampened expression of marker proteins consistent with a hypo-inflammatory phenotype, possibly indicating hampered immune responsiveness. In contrast, E2 (n = 27) showed a different immune profile characterized by increased pro-inflammatory cytokine production (IL-1β), antigen presentation (HLA-DR), phagocytosis (CD93), T-cell activation (CD25, PD1) and cytotoxic activity (Granzyme K) (**Figure 4A**).

We then investigated whether E1 and E2 were associated with different clinical features and outcomes. While upon admission vital signs and microvascular flow parameters did not differ between endotypes (**Table 1**), E1 patients were older, had more comorbidities, significantly higher C-reactive protein (CRP) and procalcitonin (PCT) levels, higher MEWS and SOFA scores, and showed more SIRS criteria (**Figure 4C**), indicating a more severe clinical course. In addition, the proportion of patients with clinical deterioration during hospitalization, defined as an increase in SOFA score, tended to be higher in E1 (36.1 vs. 14.8%, p = 0.104). Mortality was not significantly different, potentially due to the small number of events.

### 3.4 Association of distinct cellular signatures with microvascular decoupling

**Figure 5:**
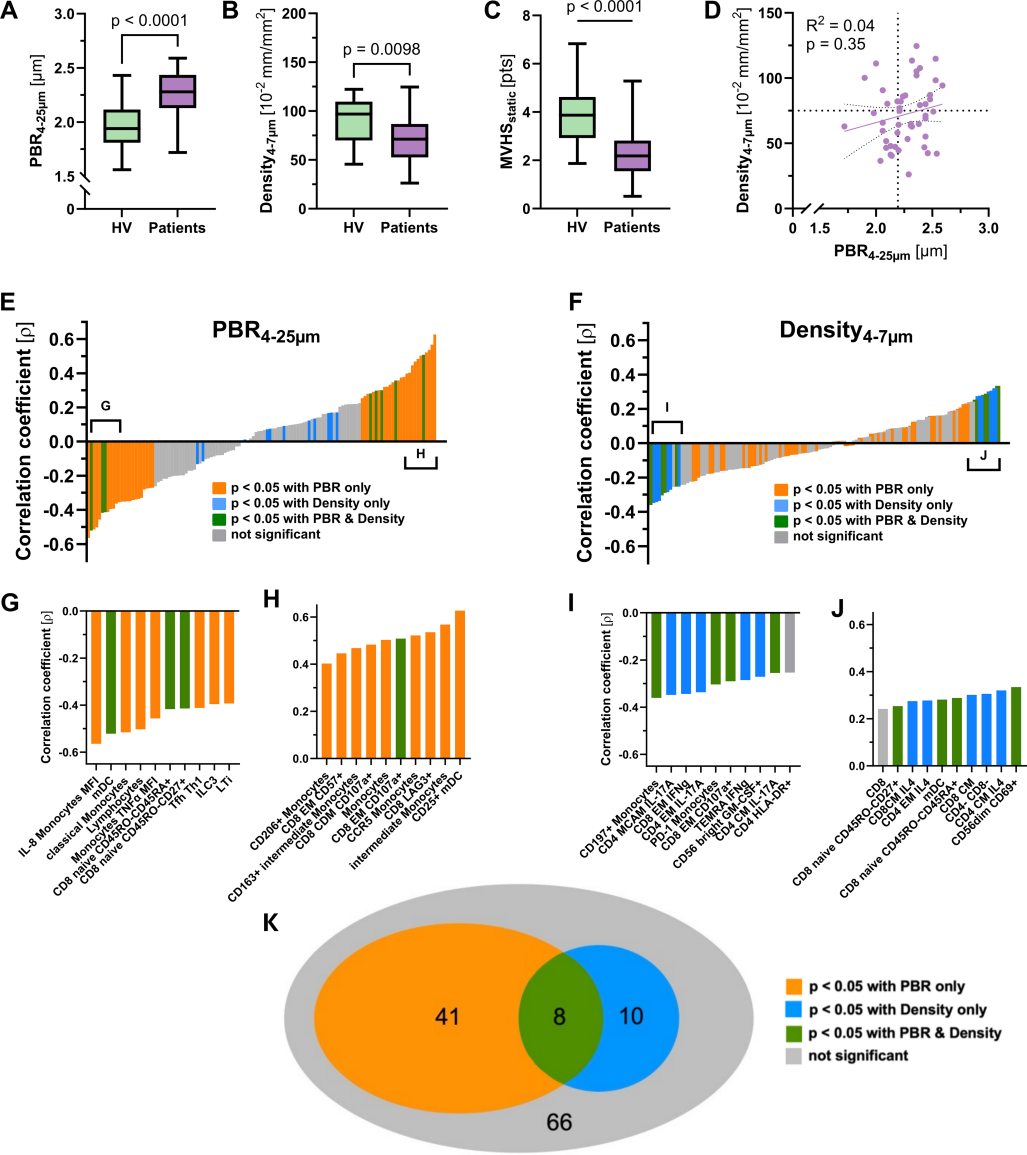
Analysis of microvascular parameters. Visualization of **(A)** PBR, **(B)** capillary density and **(C)** MVHS in Healthy volunteers and patients, respectively. **(D)** Scatter dot plots and simple linear regression (slope) with 95% confidence intervals of capillary density plotted against PBR (D4 to 25µm) in patients. The dotted lines represent the median values of capillary density and PBR, respectively. **(E, F)** show the ranked Spearman's correlation coefficients (ρ) of 125 mononuclear blood cell parameters with the perfused boundary region (PBR) and capillary density, respectively. Positive and negative correlations are shown in both panels. The colored bars represent significant correlations: orange for parameters significantly correlated with PBR, blue for those significantly correlated with capillary density, green for parameters significantly correlated with both PBR and capillary density, and grey for those with no significant correlation. **(G – J)** Insets below each panel highlight specific cell types or parameters with the most significant correlations. **(K)** Venn diagram illustrating associations of mononuclear blood cell parameters with PBR and/or capillary density.

Since microvascular impairment is considered to occur early in infection as a consequence of systemic inflammation, we asked whether distinct immune signatures may differentially correlate with capillary density and endothelial glycocalyx damage (i.e. a high PBR). Capillary density (71.2 [52.5 – 86.8] vs. 96.9 (70.0 – 109.6) 10^–2^ mm/mm^2^, p = 0.0098 and MVHS (3.87 [2.93 – 4.62] vs. 2.18 [1.55 – 2.82] pts., p < 0.001) were significantly reduced, whereas PBR (2.28 [2.13 – 2.44] vs. 1.94 [1.81 – 2.12] mm, p < 0.001) was significantly increased in ED patients, compared to healthy volunteers, respectively (**Figures 5A–C**). However, neither capillary density, nor PBR or the MVHS differed between E1 and E2 (**Supplementary Figure 2**).

We have previously shown that glycocalyx damage and capillary rarefication do neither robustly coincide, nor do they occur in proportion in every sepsis patient (7). This phenomenon of *microvascular decoupling* was also reproducible in the current group of patients with infection (R² = 0.02, p = 0.35) (**Figure 5D**).

Our next question addressed whether immune cell signatures may also reflect microvascular decoupling. Therefore, we plotted the correlation coefficients (ρ) of PBR and capillary density with each of the 72 differentially regulated parameters (and another 53 with a statistical trend) between patients and healthy volunteers (**Figures 5E, F**). Several immune cell parameters showed moderate to good correlation with PBR, with ρ values ranging from -0.56 to 0.63 (**Figures 5G, H**). In contrast, correlations with capillary density were generally weaker, with ρ values ranging from -0.36 to 0.33 (**Figure 5F**).

Colour coding of each immune cell parameter revealed mutually exclusive associations with either PBR (orange) or capillary density (blue), with only minimal overlap (green) between the two microvascular measures (**Figures 5G–K** **).**

**Figure 6:**
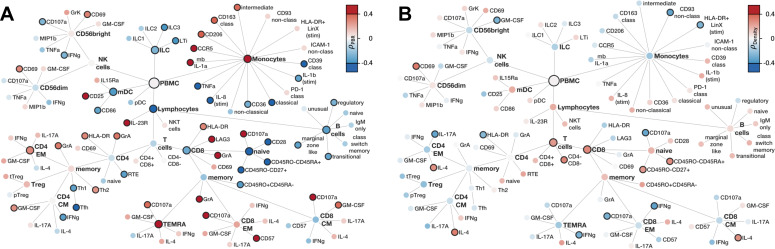
Correlation of PBMC with microvascular flow parameters **(A)** Family Tree of correlation coefficients (Spearman's ρ) of flow cytometry markers with PBR **(B)** Family Tree of correlation coefficients (Spearman's ρ) of flow cytometry markers with capillary density. Color filling represents correlation coefficients, solid node borders indicating statistical significance with p < 0.05.

To better understand the relationship between glycocalyx integrity or capillary density and associated changes in innate and adaptive immune cell signatures, the network analysis of **Figure 3** was refined by color-coding the correlation coefficient instead of the log_2_ FC (**Figure 6**). A strong association between damaged glycocalyx and cytotoxic monocytes as well as cytotoxic CD4+ and CD8+ cells was evident, while this association was not observed for capillary density.

The finding that microvascular decoupling is reflected by distinct immune signatures supports the concept that endothelial glycocalyx damage and capillary impairment are independently regulated.

## 4 Discussion

Our study shows that ED patients presenting with infection have a complex and nuanced immune profile, characterized by both activation and suppression of different immune cell subsets of the innate and adaptive immune system. Furthermore, deep immune profiling of these patients allowed the delineation of two distinct immunological endotypes: E1, which shows a suppressed immune response and higher organ damage, and E2, which is characterized by a pronounced pro-inflammatory profile and lower organ damage. Finally, the patterns of immune signatures may reflect the known microvascular decoupling present between PBR and capillary density, with PBR showing a more pronounced association with immunological changes.

To our knowledge, this is the first study to use such extensive immunophenotyping of peripheral blood to determine changes in the immune profile of patients with bacterial infections. Therefore, when interpreting the results, we can only refer to a few comparable studies of critically ill patients in the ICU who had a higher severity and longer duration of illness.

Wang et al. used flow cytometry to phenotype various immune cell subsets in PBMCs from 13 patients with septic shock (21). Consistent with our findings, they found an increased proportion of CD14+ monocytes and decreased proportions of CD4+ T cells, CD8+ T cells, NK cells and mDC cells in sepsis. They also observed an overall trend toward decreased HLA-DR expression on CD14+ monocytes in sepsis, which is an accepted surrogate marker of sepsis-induced immunosuppression (22). This finding contrasts with the significantly increased expression of HLA-DR on monocytes and CD4+ and CD8+ T cells in our ED patients.

This discrepancy suggests an early activation state of monocytes in ED patients, which is likely to decrease over time as immunosuppression becomes more pronounced in later stage ICU patients (23). However, the monocyte function appeared to be somewhat exhausted, as LPS-stimulated monocytes from patients showed slightly lower expression of e.g. TNF-α, IL-6 and IL-8 compared to healthy volunteers. Consistent with this notion, persistence of an exhausted monocyte phenotype has been reported to be associated with mortality in intensive care patients (24).

The identification of two distinct subtypes, E1 and E2, with differing immune response profiles is one key finding of our study. Notably, E1 patients exhibited a dampened expression of pro-inflammatory marker proteins, whereas E2 patients showed an enhanced immune profile characterized by increased pro-inflammatory cytokine production, antigen presentation, and cellular activation. Given these distinct immune patterns, it is conceivable that the stronger systemic inflammation seen in E1 patients is not solely due to an overwhelming infection, but rather a consequence of their impaired immune responsiveness. In contrast, E2 patients may be able to mount a more effective immune response, potentially leading to better control of the infection and reduced severity of sepsis. Unfortunately, our study design does not allow us to answer the question of whether there is a trajectory between E1 and E2, or whether these are otherwise determined groups. It is conceivable that certain ‘risk factors’ may predispose individuals to one endophenotype over the other. Some of these may be genetically determined or influenced by age and comorbidities, while others may be modifiable by targeted therapies - providing an exciting opportunity for future intervention strategies.

The delineation of these two distinct endotypes within our cohort adds granularity to existing ICU-based research, implying that different immune cell (dys-)function trajectories are not exclusive to later stages of critical illness, but already begin to diverge at earlier stages in the ED (21, 23). Furthermore, the clinical implications of these subtypes are substantial, as E1 patients tend to have a more severe clinical course, with higher levels of inflammatory markers, more comorbidities, and greater organ dysfunction, underscoring the need for deciphering immune dysfunction in this high-risk population.

Using routine clinical and laboratory data from sepsis patients on admission, Seymour et al. identified 4 clinical subphenotypes that correlated with host response patterns and clinical outcomes (25). However, the concordance with other subtypes derived from host response biomarkers and transcriptomic data is low, likely reflecting differences in clinical characteristics and underlying biology (26). Our promising data provide a starting point for considering which immune-cell markers might be added to future, larger studies.

Recent studies have described a loss of *hemodynamic coherence*, where improvements in macrohemodynamics during resuscitation are not consistently followed by subsequent changes in the microcirculation (27). This observation suggests that the microcirculation is not just a peripheral branching of vessels but is subject to differential regulation as an independent system. Taking this observation a step further, in the present study we were able to confirm the phenomenon of *decoupling* between glycocalyx damage and functional capillary density.

In two previous studies involving distinct patient cohorts and utilizing varied methodologies, we have already demonstrated that glycocalyx damage and capillary rarefaction do not consistently coincide, nor do they occur proportionally in each sepsis patient (7, 8). This phenomenon of *microvascular decoupling* was also observed among the current group of patients with infection. In other words, a patient may exhibit an intact glycocalyx despite experiencing poor red blood cell flow and capillary dropout, and vice versa. This finding suggests that the damage to the glycocalyx and the microcirculatory dysfunction may be the result of different pathological processes, rather than being a consequence of the timing or the trajectory of the injury.

While our previous study did not provide a mechanistic concept for this microvascular decoupling (7, 8), here we show for the first time that glycocalyx damage is associated with a immunological signature distinct from that of functional capillary density. This observation suggests that the eGC and the capillary perfusion in sepsis are indeed regulated by different mechanisms. In particular, the strong correlation of PBR with a relatively high number of PBMC parameters supports an active role of the immune systems in eGC degradation. This finding emphasizes that different therapeutic strategies may need to be combined to target different subsystems of the microcirculation in bacterial infection and sepsis.

At least in bacterial sepsis, the non-redundant final common pathway of eGC damage appears to be the cleavage of heparan sulfate (HS) from glycosaminoglycans by the enzyme heparanase (HPSE) (28, 29), which is released mainly from endothelial cells by pro-inflammatory cytokines such as TNF-α, IL-1β and IL-6 (14, 30, 31). HPSE-dependent damage to the glycocalyx occurs within a few minutes both *in vitro* and *in vivo*. An exciting option for inhibiting HPSE in sepsis is the administration of heparin derivatives that saturate HPSE activity as an alternative substrate. Other studies have explored the concept of endogenous sealing of a damaged glycocalyx using glycocalyx precursors such as hyaluronan, glycosaminoglycans or HS-like compounds. At least in a prophylactic setting in experimental murine sepsis, all of these approaches were able to significantly reduce end-organ damage and mortality (11). Activation of the endothelium-specific Tie2 receptor by angiopoietin-1 or Tie2 mimetics may be another exciting approach, as this pathway both blocks HPSE secretion and is thought to mediate rapid glycocalyx reassembly by providing endogenous precursors (5, 19).

The finding of an inverse correlation between the expression of TNF-α and IL-1β in monocytes with the PBR (the lower the expression, the more damaged the eGC) is therefore surprising and at first glance counterintuitive. One possible explanation is that the monocytes have already released their cytokines and are now exhausted or desensitized in terms of immunoparalysis. It is also conceivable that cytokine production is reduced by immunomodulatory, reparative processes, as suggested by a positive association of the PBR with abundance of reparative (CD206+) and anti-inflammatory (CD163+) monocytes.

Particularly surprising was the strong association of damaged eGC with the maturation of myeloid DCs (CD25+) and the concomitant activation of cytotoxic CD8+ T cell populations. Among the various mediators released, TNF-α, IL-1β and heparanase are already known to contribute to eGC degradation (30–33). Other candidates for inclusion in mechanistic experiments on glycocalyx damage are granzymes, which are positively correlated with PBR and have already been shown to induce endothelial permeability (34, 35).

There are several limitations to this study. First, the relatively small sample size and single-center design limit the generalizability of the results. However, as the advanced FACS and microvascular techniques are costly and resource intensive, much larger studies are challenging. Second, clinical heterogeneity within the cohort – including variability in infection site, timing and type of antibiotic administration, as well as pre-existing conditions and comorbidities – may have introduced additional variability in immune responses. Furthermore, we cannot exclude the possibility that demographic differences between ED patients and healthy volunteers may have influenced the results. Despite the very early collection of PBMCs from patients still in the ED, we also cannot rule out differences between endotypes due to differences in disease duration. Third, using unsupervised clustering and LASSO regression, we achieved high predictive accuracy in distinguishing between these endotypes, which appear to correlate with differences in clinical severity and inflammatory response. Although we employed a robust two-step LASSO-focused analysis with internal cross-validation and required parameter selection in at least 8 out of 10 runs to minimize overfitting, there is still a possibility that some degree of overfitting may remain, potentially limiting the generalizability of the identified endotypes. Fourth, microvascular assessment of the sublingual area may not be representative of the systemic microcirculation. However, all video recordings were made by the same person, minimizing inter-rater reliability, and the image acquisition and analysis system has been shown to provide robust and valid results in the past (6–8). Finally, we did not analyze neutrophils, nor did we perform blood cytokine arrays, proteomics or single-cell RNA sequencing, which may have provided additional insight but were beyond our budget and scope.

## 5 Conclusion

This study is the first to comprehensively characterize the immune and microvascular profiles in ED patients with infection and represents a significant advance in the field. The observed decoupling between PBR and capillary density provides new hypotheses for the interaction of the immune and microvascular systems in early infection. Our data provide a robust basis for formulating hypotheses for future mechanistic studies aimed at further unravelling the complexities of immune dysregulation and microvascular dysfunction in infection.

## Data Availability

The datasets presented in this article are not readily available because the disclosure of data is not part of the ethics application. Requests to access the datasets should be directed to Philipp.Kuempers@ukmuenster.de.
