# Supplementary material for: Changes in immune cell signatures during early infection reflect decoupling of capillary perfusion and glycocalyx dimensions
Source: Front Immunol. 2025 May 23;16:1589959. doi: 10.3389/fimmu.2025.1589959 (PMC12140986; doi:10.3389/fimmu.2025.1589959)
Supplement: Supplementary file 2 [file Presentation1.pdf]

Supplementary Material

Changes in immune cell signatures during early infection reflect decoupling of capillary perfusion and glycocalyx dimensions

Anna Hunkemöller[1†], Timo Wirth[2†], Alexandros Rovas[1], Hermann Pavenstädt[1], Luisa Klotz[2$], Philipp Kümpers*[1$]

[1]Department of Medicine D, Division of General Internal and Emergency Medicine, Nephrology, and Rheumatology, University Hospital Münster, Germany
[2]Department of Neurology with Institute of Translational Neurology, University Hospital Münster, Germany

1 Supplementary Figures and Tables

1.1 Supplementary Figures

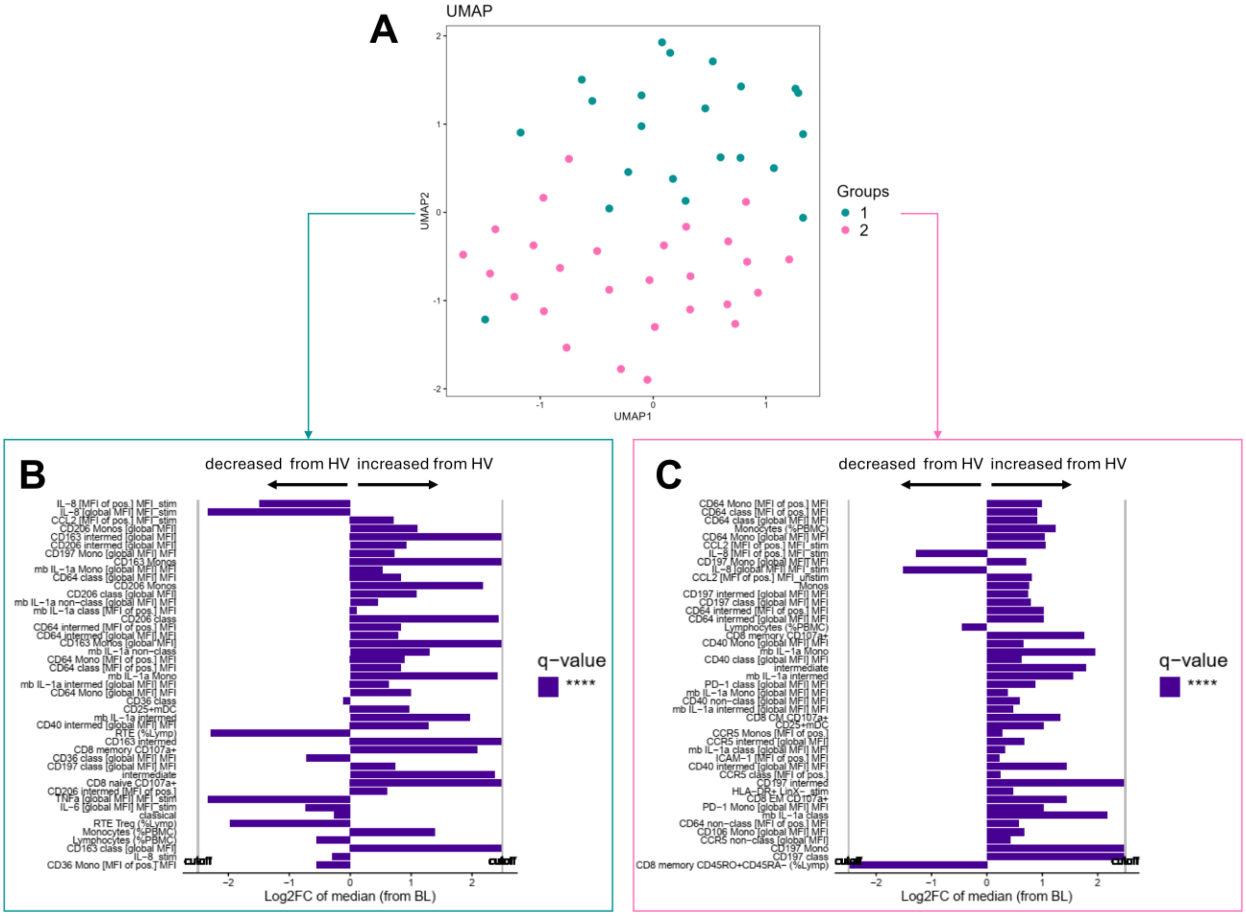

Supplementary Figure 1. Identification and hierarchical analysis of immune markers **(A)** UMAP (Uniform Manifold Approximation and Projection) plot displaying two distinct clusters based on multidimensional immune data, with Group 1 (green) and Group 2 (pink). **(B)** Bar plot showing the median Log_2 fold change (log_2 FC) of the top 10 % most significantly altered immune parameters in Group 1 compared to healthy volunteers (HV). Immune features are either increased or decreased relative to HV, with q-values represented by the intensity of the purple bars (**** indicating strong statistical significance). **(C)** Bar plot representing the median Log_2 FC of the top 10 % most significantly altered immune parameters in Group 2 compared to HV, with similar significance representation as in panel B

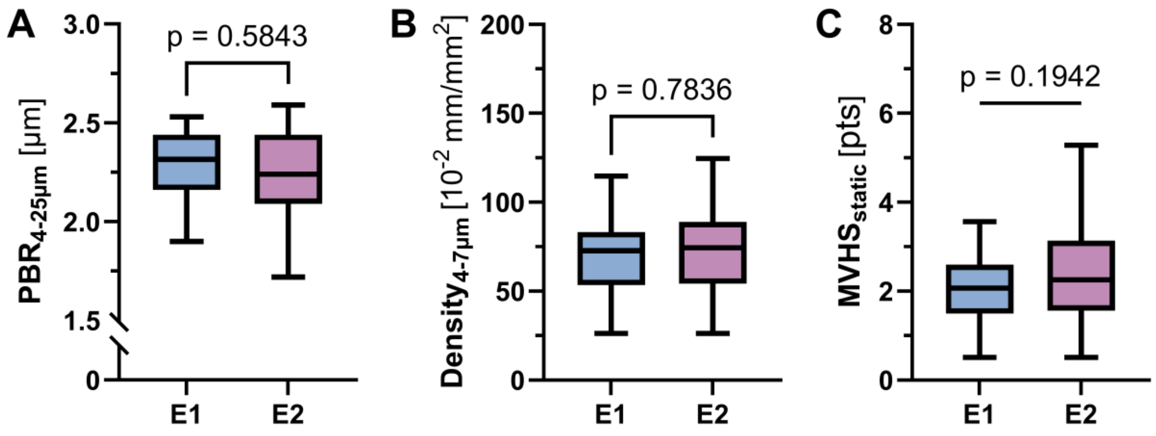

Supplementary Figure 2. Microvascular parameters in endophenotypes **(A)** Box plot showing the perfused boundary region (PBR 4−25 µm) in micrometers for E1 (blue) and E2 (purple), with p-values indicated. **(B)** Box plot illustrating glycocalyx density (Density 4−7 µm) in 10^{-2} mm/mm² for E1 and E2. **(C)** Box plot representing the microvascular health score ($MVHS_{static}$) in points for the two groups

1.2 Supplementary Tables

Supplementary Table 1. Gating Strategies. IL: interleukin; CD: cluster of differentiation; IFN-γ: interferon gamma; GM-CSF: granulocyte macrophage colony stimulating factor

**Population name**	**Gating Strategy**
Lymphocytes	FSC vs. SSC
Monocytes	CD14+ PBMCs
Classical monocytes	CD14high CD16- Monocytes
Intermediate monocytes	CD14high CD16+ Monocytes
Non-classical monocytes	CD14+ CD16high Monocytes
T cells	CD3+ CD56- Lymphocytes
NKT cells	CD3+CD56+ Lymphocytes
double negative	CD4-CD8- T cells
double positive	CD4+CD8+ T cells

Va24-Ja18+ NKT	Va24-Ja18+ NKT cells
CD4/CD8 CD69+	CD69+ CD4/CD8 Tcells
C56dim/CD56bright CD69+	CD69+ C56dim/CD56bright NK cells
CD4/CD8 HLA-DR+	HLA-DR+ CD4/CD8 Tcells
C56dim/CD56bright HLA-DR+	HLA-DR+ C56dim/CD56bright CD3- CD56+ NK cells
B cells	CD19+ CD20+ Lymphocytes
B naive	CD27- IgD+ IgM+ B cells
B class switch memory	CD27+ IgD- IgM- B cells
B marginal zone like	CD27+ IgD+ IgM+ B cells
B IgM only	CD27+ IgD- IgM+ B cells
B unusual	CD21+ CD38- IgD+ IgM+ B cells
B transitional	CD21+ CD38+ CD24+ IgD+ IgM+ B cells
B regulatory	CD27+ CD24+ CD38+ B cells
CD4	CD4+ CD8- T cells
CD8	CD8+ CD4- T cells
CD4/CD8 naive	CD45RO- CD45RA+ CD4+ or CD8+ T cells
Recent Thymic Emigrant (RTE) cells	CD31+ naive CD4 cells
CD4/CD8 memory	CD45RO+ CD45RA- CD4+ or CD8+ T cells
CD4/CD8 central memory	CD45RO+ CD27+ CD4+ or CD8+ T cells
CD4/CD8 effector memory	CD45RO+ CD27- CD4+ or CD8+ T cells
CD4/CD8 CD27+ naive	CD45RO- CD27+ CD4+ or CD8+ T cells
CD4/CD8 TEMRA	CD45RO- CD27- CD4+ or CD8+ T cells
Th1	CD45RO+ CCR4- CCR6- CD183+ CD4+ T cells
Th2	CD45RO+ CCR4+ CCR6- CD183- CD4+ T cells
Th17	CD45RO+ CCR4+ CCR6+ CD183- CD4+ T cells
Th17 MCAM	CD146+ Th17 cells
Memory MCAM	CD45RO+ CD146+ CD4+ T cells
T follicular helper (Tfh)	CD45RO+ CXCR5+ PD1+ ICOS+ CD4+ T cells
Tfh Th1	CXCR5+ PD1+ ICOS+ Th1 cells
Tfh Th2	CXCR5+ PD1+ ICOS+ Th2 cells
Tfh Th17	CXCR5+ PD1+ ICOS+ Th17 cells
CD8 MAIT cells	CD161+ CD8 T cells
CD4/CD8 memory GM-CSF	CD45RO+ GM-CSF+ CD4+ or CD8+ cells
CD4/CD8 central memory GM-CSF	CD45RO+ CD27+ GM-CSF+ CD4+ or CD8+ cells
CD4/CD8 effector memory GM-CSF	CD45RO+ CD27- GM-CSF+ CD4+ or CD8+ cells
CD4/CD8 memory IFN-g	CD45RO+ IFN-g+ CD4+ or CD8+ cells
CD4/CD8 central memory IFN-g	CD45RO+ CD27+ IFN-g+ CD4+ or CD8+ cells
CD4/CD8 effector memory IFN-g	CD45RO+ CD27- IFN-g+ CD4+ or CD8+ cells
CD4/CD8 memory IL-17A	CD45RO+ IL-17A+ CD4+ or CD8+ cells
CD4/CD8 central memory IL-17A	CD45RO+ CD27+ IL-17A+ CD4+ or CD8+ cells
CD4/CD8 effector memory IL-17A	CD45RO+ CD27- IL-17A+ CD4+ or CD8+ cells
CD4/CD8 memory IL-22	CD45RO+ IL-22+ CD4+ or CD8+ cells
CD4/CD8 central memory IL-22	CD45RO+ CD27+ IL-22+ CD4+ or CD8+ cells

CD4/CD8 effector memory IL-22	CD45RO+ CD27- IL-22+ CD4+ or CD8+ cells
CD4/CD8 memory IL-4	CD45RO+ IL-4+ CD4+ or CD8+ cells
CD4/CD8 central memory IL-4	CD45RO+ CD27+ IL-4+ CD4+ or CD8+ cells
CD4/CD8 effector memory IL-4	CD45RO+ CD27- IL-4+ CD4+ or CD8+ cells
CD4/CD8 memory TNF-a	CD45RO+ TNF-a+ CD4+ or CD8+ cells
CD4/CD8 central memory TNF-a	CD45RO+ CD27+ TNF-a+ CD4+ or CD8+ cells
CD4/CD8 effector memory TNF-a	CD45RO+ CD27- TNF-a+ CD4+ or CD8+ cells
TEMRA GM-CSF	CD45RO-CD27- GM-CSF+ CD8+ T cells
TEMRA IFN-g	CD45RO-CD27- IFN-g+ CD8+ T cells
TEMRA IL-17A	CD45RO-CD27- IL-17A+ CD8+ T cells
TEMRA IL-22	CD45RO-CD27- IL-22+ CD8+ T cells
TEMRA IL-4	CD45RO-CD27- IL-4+ CD8+ T cells
TEMRA TNF-a	CD45RO-CD27- TNF-a+ CD8+ T cells
Regulatory T cells (Treg)	CD45RO+ Foxp3+ CD25+ CD127low CD4+ Tcells
pTreg	CD45RO+ Foxp3+ CD25+ CD127low Helios- CD4+ Tcells
tTreg	CD45RO+ Foxp3+ CD25+ CD127low Helios+ CD4+ Tcells
RTE-like cells	CD45RO- CD31+ CD4+ T cells
RTE Treg	CD45RO- CD31+ Foxp3+ CD127low CD4+ T cells
Degranulating NK bright	CD107a+ CD56bright CD3- CD56+ NK cells
Degranulating NK dim	CD107a+ CD56dim NK cells
Degranulating NK dim CD57+	CD107a+ CD57+ CD56dim NK cells
Degranulating NK dim NKG2C+	CD107a+ NKG2C+ CD56dim NK cells
Degranulating NK dim CD57+ NKG2C+	CD107a+ CD57+ NKG2C+ CD56dim NK cells
NK bright IFN-g	IFN-g+ CD56bright NK cells
NK bright MIP1-b	MIP1-b+ CD56bright NK cells
NK bright TNF-a	TNF-a+ CD56bright NK cells
NK dim IFN-g	IFN-g+ CD56dim NK cells
NK dim MIP1-b	MIP1-b+ CD56dim NK cells
NK dim TNF-a	TNF-a+ CD56dim NK cells
CTL dim CD57+	CD57+ CD56dim NK cells
NK dim memory cells	CD57+ NKG2C+ FcER1g-Syk- CD56 dim NK cells
Degranulating CD8 naive	CD45RO- CD27+ CD107a+ CD8+ T cells
Degranulating CD8 memory	CD45RO+ CD107a+ CD8+ T cells
Degranulating CD8 central memory	CD45RO+ CD27- CD107a+ CD8+ T cells
Degranulating CD8 effector memory	CD45RO+ CD27+ CD107a+ CD8+ T cells
Degranulating TEMRA	CD45RO- CD27- CD107a+ CD8+ T cells
NK bright Granzyme K	CD56bright CD16dim/- CD3- GrK+
NK dim Granzyme K	CD56dim CD16bright CD3- GrK+
CD4 memory Granzyme K	CD45RO+ GrK+ CD4+ T cells
CD8 memory Granzyme K	CD45RO+ GrK+ CD8+ T cells
myeloid dendritic cells (mDCs)	Lin(CD3/CD19/CD56)- HLA-DR+ SSClow CD14- CD11c+ CD1c+
CD141+ mDCs	Lin(CD3/CD19/CD56)- HLA-DR+ SSClow CD14- CD11c+ CD1c- CD141+

plasmacytoid dendritic cells (pDCs)	Lin(CD3/CD19/CD56)- HLA-DR+ SSClow CD14- CD11c- CD303+
CD86+ mDC	CD86+ mDCs
CD86+ CD141+	CD86+ CD141+ mDCs
CD86+ pDC	CD86+ pDCs
CD25+ mDC	CD25+ mDCs
Innate lymphoid cells (ILCs)	CD45+ CD34- Lin(CD3/CD14/CD19/CD20)- CD123- CD11c-
NK cells	CD45+ CD34- Lin(CD3/CD14/CD19/CD20)- CD123- CD11c- CD56+
CD56bright NK cells	CD56bright CD16dim/- NK cells
CD56dim NK cells	CD56dim CD16bright NK cells
ILC1	CD56- NKp46- CD117- CRTH2- ILCs
ILC2	CD56- NKp46- CD117- CRTH2+ ILCs
ILC3	CD56dim NKp46+ CD117+ ILCs
Lymphoid tissue inducer cells (LTi)	CD56- NKp46- CD117+ ILCs
Hematopoietic stem cells (HSCs)	CD34+ CD45- PBMCs
Immature/classical Monocytes	CD14+ HLA-DR+ CD16- CD192high Mononuclear cells
Intermediate CCR2+ Monocytes	CD14+ HLA-DR+ CD16+ CD192+ Mononuclear cells
Mature/non-classical Monocytes	CD14+ HLA-DR+ CD16high CD192- Mononuclear cells
Myeloid derived suppressor cells (MDSCs)	CD14+ HLA-DR+ CD16- CD124+ Mononuclear cells
M1-like monocytes	CX3CR1+ CCR5+ Monocytes
M2-like monocytes	CD206+ Monocytes
M2-like CD121b+ monocytes	CD121b+ M2-like monocytes
CD80 monocytes	CD80+ Monocytes
CD86 monocytes	CD86+ Monocytes
CD80 monocytes	CD80+ Monocytes
CD86 monocytes	CD86+ Monocytes
CalgranB monocytes	CalgranulinB+ monocytes
CD93 monocytes	CD93+ monocytes
CD163 monocytes	CD163+ monocytes
CX3CR1 monocytes	CX3CR1+ monocytes
CD121b monocytes	CD121b+ monocytes
CD39 monocytes	CD39+ monocytes
CCR5 monocytes	CCR5+ monocytes
CCR7 monocytes	CCR7+ monocytes
CD36 monocytes	CD36+ monocytes
PD-1 monocytes	PD-1+ monocytes
CD106 monocytes	CD106+ monocytes
CD124 monocytes	CD124+ monocytes
CD31 monocytes	CD31+ monocytes
membrane bound IL-1 alpha monocytes	mbIL-1a+ monocytes
TIM-3 monocytes	TIM-3+ monocytes
CD69 monocytes	CD69+ monocytes

ICAM1 monocytes	ICAM1+ monocytes
CD40 monocytes	CD40+ monocytes
CD68 monocytes	CD68+ monocytes
CD64 monocytes	CD64+ monocytes

Supplementary Table 2. List of Antibodies Used for Flow Cytometric Analysis

Antibody	Clone	Company
Calgranulin B	27E10	ImmunoTools
CD1c / BDCA-1	AD5-8E7	Miltenyi
CD25	7G7B6	Ancell
CD36	5-271	Biolegend
CD45RA	ALB11	Beckman Coulter
CD69	FN50	Miltenyi
CD107a	H4A3	BD Biosciences
CD158a/h (KIR2DL1/S1/S3/S5)	HP-MA4	Biolegend
CD158b/j (KIR2DL2/L3)	DX27	Biolegend
CD158e1/2 (KIR3DL1/S1)	REA168	Miltenyi
CD158i (KIR2DS4)	180704	R&D Systems
CD183/CXCR3	G025H7	Biolegend
CX3CR1	2A9-1	Biolegend
FceR1 gamma	polyclonal	Merck Millipore
Granzyme K	GM6C3	SantaCruz
Helios	22F6	Biolegend
INFγ	4S.B3	Biolegend
IgD	IA6-2	Biolegend
anti-membrane IL-1 alpha / IL-1F1 Membrane Form		R&D Systems
IL-17A	BL168	Biolegend
Lin-3 (CD3/14/19/20)	Sk7 (CD3) SJ25C1 (CD19) L27 (CD20) MφP9 (CD14)	BD Biosciences
CD34	581	Biolegend
CD54 (ICAM-1)	HA58	Biolegend
CD57	REA769	Miltenyi
CD69	TP1.55.3	Beckman Coulter
CD80	L307.4	BD Biosciences
CD93	VIMD2	Biolegend
CD159c/NKG2C	REA205	Miltenyi
CD252 / OX40L	11C3.1	Biolegend
CD279 / PD1	eBioJ105	eBioscience
FoxP3	PCH101	eBioscience
Granzyme B	REA226	Miltenyi
IL-15Ra (CD215)	eBioJM7A4	eBioscience

IL-22	22URTI	eBioscience
IL-23R	218213	R&D Systems
MCP-1 (CCL2)	2H5	Biolegend
TNFα	MAb11	Biolegend
CD4	SFCI12T4D11	Beckman Coulter
CD16	3G8	Beckman Coulter
CD24	ALB9	Beckman Coulter
CD31	WM59	Biolegend
CD56	N901	Beckman Coulter
CD137	REA765	Miltenyi
CD337 / NKp30	REA823	Miltenyi
HLA-DR	Immu-357	Beckman Coulter
CD3	UCHT1	Beckman Coulter
CD31	WM59	Biolegend
CD38	HIT2	Biolegend
CD40	5C3	invitrogen
CD45RO	UCHL1	Biolegend
CD86	HA5.2B7	Beckman Coulter
CD106	51-10C9	BD Biosciences
CD163	GHI/61	Biolegend
CD194/CCR4	L291H4	Biolegend
CD335/NKp46	9E2	Biolegend
IL-6	MQ2-13A5	Biolegend
TNFa	Mab11	Biolegend
Va24-Ja18	6B11	Biolegend
CD1c/BDCA-1	L161	Biolegend
CD27	1A4CD27	Beckman Coulter
CD56	N901 (HLDA6)	Beckman Coulter
CD123	6H6	Biolegend
CD159a/NKG2A	Z199	Beckman Coulter
CD196/CCR6	11A9	BD Biosciences
CX3CR1	2A9-1	Biolegend
Granzyme A	CB9	eBioscience
INFγ	4S.B3	Biolegend
LAG3 / CD223	11C3C65	Biolegend
TIGIT	MBSA43	eBioscience
CD3	Hit3a	Biolegend
CD4	13B8.2	Beckman Coulter
CD19	HIB19	Biolegend
CD23	EBVCS-5	Biolegend
CD56	AF12-7H3	Miltenyi
CD68	Y1/82A	Biolegend
CD121b / IL-1R2	REA744	Miltenyi
CD124/IL-4R	G077F6	Biolegend
CD226/DNAM-1	11A8	Biolegend
CD336/NKp44	P44-8	Biolegend

GM-CSF	BVD2-21C11	Miltenyi
Granzyme M	4B2G4	eBioscience
IL-8	E8N1	Biolegend
Streptavidin		Biolegend
SYK	4D10.1	eBioscience
TIM3	F38-2E2	Miltenyi
CD8	B9.11	Beckman Coulter
CD11c	Bu15	Biolegend
CD14	M5E2	Biolegend
CD19	J3-119	Beckman Coulter
CD28	CD28.2	Biolegend
CD127	R34.34	Beckman Coulter
CD3	HIT3a	Biolegend
CD3	UCHT1	Beckman Coulter
CD16	3G8	Beckman Coulter
CD19	HIB19	Biolegend
CD20	2H7	Biolegend
CD56	5.1H11	Biolegend
CD66b	G10F5	Biolegend
CD141	AD5-14H12	Miltenyi
CD244 / 2B4	C1.7	Biolegend
CD294/CRTH2	BM16	Biolegend
CD8	B9.11	Beckman Coulter
CD16	3G8	Beckman Coulter
CD21	HB5	Miltenyi
CD31	WM59	Biolegend
CD39	A1	Biolegend
CD45RO	UCHL1	Biolegend
CD57	QA17A04	Biolegend
CD64	10.1	Biolegend
CD253	RIK-2	BD Biosciences
CD278 / ICOS	C398.4A	Biolegend
CD303/BDCA-2	AC144	Miltenyi
GM-CSF	BVD2-21C11	BD Biosciences
IL-1β	H1b-98	Biolegend
Mip-1ß	D21-1351	BD Biosciences
Perforin	delta G9	Miltenyi
CD3	OKT3	Biolegend
CD4	OKT4	Biolegend
CD8	SK1	Biolegend
CD14	M5E2	Biolegend
CD18	L130	BD Biosciences
CD117/cKit	104D2	Biolegend
CD146	P1H12	BD Biosciences
CD314/NKG2D	1D11	Biolegend

HLA-DR	L243	Biolegend
IgM	MHM-88	Biolegend
CD45	HI30	Biolegend
CD45RO	UCHL1	Biolegend
CD56 / NCAM	HCD56	Biolegend
CD195	2D7/CCR5	BD Biosciences
CD197 / CCR7	G043H7	Biolegend
CD3	UCHT1	Biolegend
CD25	BC96	Biolegend
CD27	O323	Biolegend
CD127 / IL-7Ra	A019D5	Biolegend
CD192/CCR2	LS1321D9	BD Biosciences
CD4	Okt4	Biolegend
CD32	FLI8.26	BD Biosciences
CD62L	DREG-56	Biolegend
CD161	HP-3G10	Biolegend
CD197 / CCR7	G043H7	Biolegend
CD206	15-2	Biolegend
IL4	MP4-25D2	BD Biosciences
TNFa	Mab11	Biolegend
CD3	OKT3	Biolegend
CD244 / 2B4	eBioC1.7	eBioscience
CD226 / DNAM	DX11	BD Biosciences
CD185 / CXCR5	J252D4	Biolegend

Supplementary Table 3. List of Fluorochromes Used for Flow Cytometric Analysis

**Fluorochrome**	**Name**	**Clone**	**Isotype**	**Company**
FITC	Calgranulin B	27E10	mIgG1,k	ImmunoTools
FITC	CD1c / BDCA-1	AD5-8E7	mIgG2a	Miltenyi
FITC	CD25	7G7B6	mIgG2a	Ancell
FITC	CD36	5-271	mIgG2a,k	Biolegend
FITC	CD45RA	ALB11	mIgG1	Beckman Coulter
FITC	CD69	FN50	mIgG1,k	Miltenyi
FITC	CD107a	H4A3	mIgG1,k	BD Biosciences
FITC	CD158a/h (KIR2DL1/S1/S3/S5)	HP-MA4	mIgG2b, k	Biolegend
FITC	CD158b/j (KIR2DL2/L3)	DX27	mIgG2a	Biolegend
FITC	CD158e1/2 (KIR3DL1/S1)	REA168	recombinant human IgG1	Miltenyi
FITC	CD158i (KIR2DS4)	180704	mIgG2b	R&D Systems
FITC	CD183/CXCR3	G025H7	mIgG1	Biolegend

FITC	CX3CR1	2A9-1	rIgG2b,k	Biolegend
FITC	FceR1 gamma	polyclonal	rabbit IgG	Merck Millipore
FITC	Granzyme K	GM6C3	mIgG2b, k	SantaCruz
FITC	Helios	22F6	Armenian hamster IgG	Biolegend
	INFγ	4S.B3	mIgG1,k	Biolegend
	IgD	IA6-2	mIgG2a	Biolegend
FITC	anti-membrane IL-1 alpha / IL-1F1 Membrane Form		Mouse IgG1	R&D Systems
FITC	IL-17A	BL168	mIgG1,k	Biolegend
FITC	Lin-3 (CD3/14/19/20)	Sk7 (CD3) SJ25C1 (CD19) L27 (CD20) MφP9 (CD14)	mIgG1, k (CD3, CD19, CD20) mIgG2b, k (CD14)	BD Biosciences
PE	CD34	581	mIgG1, k	Biolegend
PE	CD54 (ICAM-1)	HA58	mIgG1,k	Biolegend
PE	CD57	REA769	recombinant hIgG1	Miltenyi
PE	CD69	TP1.55.3	mIgG2b	Beckman Coulter
PE	CD80	L307.4	C3H, mIgG1	BD Biosciences
PE	CD93	VIMD2	mIgG1,k	Biolegend
PE	CD159c/NKG2C	REA205	recombinant hIgG1	Miltenyi
PE	CD252 / OX40L	11C3.1	mIgG1, k	Biolegend
PE	CD279 / PD1	eBioJ105	mIgG1, k	eBioscience
PE	FoxP3	PCH101	rIgG2a	eBioscience
PE	Granzyme B	REA226	recombinant hIgG1	Miltenyi
PE	IL-15Ra (CD215)	eBioJM7A4	mIgG2b	eBioscience
PE	IL-22	22URTI	mIgG1, k	eBioscience
PE	IL-23R	218213	mIgG2b	R&D Systems
PE	MCP-1 (CCL2)	2H5	Armenian Hamster IgG	Biolegend
PE	TNFα	MAb11	mIgG1, k	Biolegend
ECD	CD4	SFCI12T4D11	mIgG1	Beckman Coulter
ECD	CD16	3G8	mIgG1	Beckman Coulter
ECD	CD24	ALB9	mIgG1	Beckman Coulter
PE-Dazzle594	CD31	WM59	mIgG1, k	Biolegend
ECD	CD56	N901	mIgG1	Beckman Coulter
PE-Vio615	CD137	REA765	recombinant hIgG1	Miltenyi
PE-Vio615	CD337 / NKp30	REA823	recombinant hIgG1	Miltenyi
ECD	HLA-DR	Immu-357	mIgG1	Beckman Coulter
PE-Cy5.5	CD3	UCHT1	mIgG1	Beckman Coulter
PerCP-Cy5.5	CD31	WM59	mIgG1, k	Biolegend
PerCP-Cy5.5	CD38	HIT2	mIgG1, k	Biolegend

PerCP eF710	CD40	5C3	mIgG1,k	invitrogen
PerCP-Cy5.5	CD45RO	UCHL1	IgG2a, κ	Biolegend
PE-Cy5.5	CD86	HA5.2B7	mIgG2b	Beckman Coulter
PerCP-Cy5.5	CD106	51-10C9	m IgG1, κ	BD Biosciences
PerCP/Cy5.5	CD163	GHI/61	mIgG1, k	Biolegend
PerCP-Cy5.5	CD194/CCR4	L291H4	mIgG1, k	Biolegend
PerCP-Cy5.5	CD335/NKp46	9E2	mIgG1, k	Biolegend
PerCP-Cy5.5	IL-6	MQ2-13A5	rIgG1,k	Biolegend
PerCP-Cy5.5	TNFa	Mab11	mIgG1,k	Biolegend
PerCP-Cy5.5	Va24-Ja18	6B11	mIgG1,k	Biolegend
PE-Cy7	CD1c/BDCA-1	L161	mIgG1, k	Biolegend
PE-Cy7	CD27	1A4CD27	mIgG1	Beckman Coulter
PC7	CD56	N901 (HLDA6)	mIgG1	Beckman Coulter
PE-Cy7	CD123	6H6	mIgG1, k	Biolegend
PE-Cy7	CD159a/NKG2A	Z199	mIgG2b	Beckman Coulter
PE-Cy7	CD196/CCR6	11A9	mIgG1, k	BD Biosciences
PE-Cy7	CX3CR1	2A9-1	rIgG2b, κ	Biolegend
PE-Cy7	Granzyme A	CB9	mIgG1, k	eBioscience
PE-Cy7	INFy	4S.B3	mIgG1, k	Biolegend
PE-Cy7	LAG3 / CD223	11C3C65	mIgG1, k	Biolegend
PE-Cy7	TIGIT	MBSA43	mIgG1, k	eBioscience
APC	CD3	Hit3a	mIgG2a, κ	Biolegend
APC	CD4	13B8.2	mIgG1	Beckman Coulter
APC	CD19	HIB19	mIgG1, k	Biolegend
APC	CD23	EBVCS-5	mIgG1, k	Biolegend
APC	CD56	AF12-7H3	mIgG1, k	Miltenyi
AF647	CD68	Y1/82A	mIgG2b,k	Biolegend
APC	CD121b / IL-1R2	REA744	recombinant hIgG1	Miltenyi
APC	CD124/IL-4R	G077F6	mIgG2a	Biolegend
APC	CD226/DNAM-1	11A8	mIgG1, k	Biolegend
APC	CD336/NKp44	P44-8	mIgG1, k	Biolegend
APC	GM-CSF	BVD2-21C11	rIgG2a, k	Miltenyi
eFluor660	Granzyme M	4B2G4	mIgG1, k	eBioscience
APC	IL-8	E8N1	mIgG1, k	Biolegend
APC	Streptavidin			Biolegend
APC	SYK	4D10.1	mIgG2a, k	eBioscience
APC	TIM3	F38-2E2	mIgG1κ	Miltenyi
APC-A700	CD8	B9.11	mIgG1	Beckman Coulter
AF700	CD11c	Bu15	mIgG1, k	Biolegend
AF700	CD14	M5E2	mIgG2a, k	Biolegend
APC-A700	CD19	J3-119	mIgG1	Beckman Coulter
AF700	CD28	CD28.2	mIgG1, κ	Biolegend
APC-A700	CD127	R34.34	mIgG1	Beckman Coulter
APC/Cy7	CD3	HIT3a	mIgG2a,k	Biolegend

APC-A750	CD3	UCHT1	mIgG1, k	Beckman Coulter
APC-A750	CD16	3G8	mIgG1	Beckman Coulter
APC/Cy7	CD19	HIB19	mIgG1,k	Biolegend
APC-Fire750	CD20	2H7	mIgG2b, κ	Biolegend
APC/Cy7	CD56	5.1H11	mIgG1,k	Biolegend
APC/Cy7	CD66b	G10F5	mIgM,k	Biolegend
APC-Vio770	CD141	AD5-14H12		Miltenyi
APC/Cy7	CD244 / 2B4	C1.7	mIgG1, k	Biolegend
APC/Cy7	CD294/CRTH2	BM16	rIgG2a, k	Biolegend
PacB	CD8	B9.11	mIgG1	Beckman Coulter
PacB	CD16	3G8	mIgG1	Beckman Coulter
VioBlue	CD21	HB5	mIgG2a, k	Miltenyi
BV421	CD31	WM59	mIgG1, k	Biolegend
BV421	CD39	A1	mIgG1, k	Biolegend
BV421	CD45RO	UCHL1	mIgG2a	Biolegend
BV421	CD57	QA17A04	mIgG1,k	Biolegend
PacB	CD64	10.1	mIgG1,k	Biolegend
BV421	CD253	RIK-2	mIgG1	BD Biosciences
BV421	CD278 / ICOS	C398.4A	Armenian Hamster IgG	Biolegend
VioBlue	CD303/BDCA-2	AC144	mIgG1, k	Miltenyi
BV421	GM-CSF	BVD2-21C11	rat Lewis IgG2a	BD Biosciences
PacB	IL-1β	H1b-98	mIgG1, k	Biolegend
V-450	Mip-1ß	D21-1351	mIgG1, κ	BD Biosciences
VioBlue	Perforin	delta G9	mIgG2b, κ	Miltenyi
BV510	CD3	OKT3	mIgG2a, k	Biolegend
BV510	CD4	OKT4	mIgG2b	Biolegend
BV510	CD8	SK1	mIgG1, k	Biolegend
BV510	CD14	M5E2	mIgG2a, k	Biolegend
BV510	CD18	L130	BALB/c mIgG1, κ	BD Biosciences
BV510	CD117/cKit	104D2	mIgG1, k	Biolegend
BV510	CD146	P1H12	mIgG1, κ	BD Biosciences
BV510	CD314/NKG2D	1D11	mIgG1, κ	Biolegend
BV510	HLA-DR	L243	mIgG2a, k	Biolegend
BV510	IgM	MHM-88	mIgG1, κ	Biolegend
BV605	CD45	HI30	mIgG1,k	Biolegend
BV605	CD45RO	UCHL1	mIgG2a,k	Biolegend
BV605	CD56 / NCAM	HCD56	mIgG1,k	Biolegend
BV605	CD195	2D7/CCR5	m C57BL/6 IgG2a, κ	BD Biosciences
BV605	CD197 / CCR7	G043H7	mIgG2a,k	Biolegend
BV650	CD3	UCHT1	mIgG1,k	Biolegend
BV650	CD25	BC96	mIgG1,k	Biolegend
BV650	CD27	O323	mIgG1,k	Biolegend
BV650	CD127 / IL-7Ra	A019D5	mIgG1,k	Biolegend

BV650	CD192/CCR2	LS1321D9	Mouse BALB/c IgG2a, κ	BD Biosciences
BV785	CD4	Okt4	mIgG2b,k	Biolegend
BV786	CD32	FLI8.26	Mouse BALB/c IgG2b, κ	BD Biosciences
BV785	CD62L	DREG-56	mIgG1,k	Biolegend
BV785	CD161	HP-3G10	mIgG1,k	Biolegend
BV785	CD197 / CCR7	G043H7	mIgG2a, k	Biolegend
BV785	CD206	15-2	mIgG1,k	Biolegend
BV786	IL4	MP4-25D2	rIgG1	BD Biosciences
BV785	TNFa	Mab11	mIgG1,k	Biolegend
purified	CD3	OKT3	mIgG2a, κ	Biolegend
purified	CD244 / 2B4	eBioC1.7	mIgG1, κ	eBioscience
purified	CD226 / DNAM	DX11	BALB/c mIgG1, κ	BD Biosciences
Biotin	CD185 / CXCR5	J252D4	mIgG1, k	Biolegend
